# A Fast and Simple Method for the Determination of TBA in ^18^F-Labeled Radiopharmaceuticals

**DOI:** 10.3390/ph13020027

**Published:** 2020-02-11

**Authors:** Nils Erik Halvorsen, Ole Heine Kvernenes

**Affiliations:** Center for Nuclear medicine/PET, Department of Radiology, Haukeland University Hospital, Jonas Lies vei 65, N-5021 Bergen, Norway; nils.erik.halvorsen@helse-bergen.no

**Keywords:** tetrabutylammonium, TBA, PET, quality control, spot test, radiopharmaceuticals, PSMA, FDOPA, FDG, iodoplatinate

## Abstract

A simple color spot test for determining the presence of residual tetrabutylammonium (TBA) in various ^18^F-radiopharmaceuticals is described. The test can be performed in less than five minutes. Iodoplatinate-saturated TLC plates are initially spotted with the ^18^F-radiopharmaceutical to be tested and TBA, then with deionized water or hydrogen peroxide (H_2_O_2_) solution, depending on if an antioxidant stabilizer is part of the pharmaceutical matrix. A distinct brown spot is visible at TBA concentrations of 50 µg/mL and up.

## 1. Introduction

Tetrabutylammonium (TBA) is a quaternary ammonium cation often used as a phase-transfer catalyst in the ^18^F-labeling reactions of many common ^18^F-radiopharmaceuticals. Due to TBA being labeled as a specified impurity in Pharm Eur. ^18^F-radiopharmaceutical monographs, residual TBA in the finished pharmaceutical must be determined before release and use [1]. In Pharm Eur., the limit for TBA is ≤ 2.6 mg/V. V is defined as the maximum injected dose, in milliliters [1], and this volume defines the final quality control (QC) limits before release. Each producer is free to set V according to their own production and needs, and typically V is often set in the region between 10–20 mL, depending on the radiotracer and on the production site. This value should not be confused with the final production volume. For [^18^F]FLT, [^18^F]PSMA, and [^18^F]FDOPA, we have chosen V = 10. The implications of this are that the maximum limit of TBA should be ≤ 0.26 mg/mL, in our QC of [^18^F]FLT. If V was set to 20mL, the maximum limit of TBA would be ≤ 0.13 mg/mL.

The method for determining TBA content described in Pharm Eur. is based on ion-pairing HPLC [1]. Some shortcomings of this method and possible workarounds have been mentioned in the literature in recent times [2,3], and although modified methods work for determining TBA in [^18^F]FLT, ion-pairing HPLC offers some significant drawbacks, like very long equilibration times for stable peak areas (12 h in our hands), which means it is almost a necessity to have two HPLC-systems for routine quality control when using this methodology. A further complication is that ion-pairing HPLC does not seem to work (no peak for TBA is observed) when analyzing TBA in more complex ^18^F-radiopharmaceutical formulations, like [^18^F]PSMA-1007 and [^18^F]FDOPA, most likely due to matrix effects disturbing the ion-pairing relationship. Other described procedures for determining TBA are based on treating or eluting TLC-spots with MeOH/NH_4_OH and staining with iodine vapors [4,5], but these procedures are, in our experience, time-consuming due to long TLC elution times (10–15 min) and hard to consistently reproduce because of the many variables (application of the spot, drying of the plate, amount of iodine vapor, time in the iodine chamber) that can affect the outcome of the test. We have been able to use the iodine staining methods to visualize TBA with no antioxidant present in the matrix. However, when an antioxidant is present, these tests have been unable to produce a spot for TBA in our hands. Lastly, some new, quantitative methods for determining TBA have been proposed [6]. Of these methods, the spectrophotometric method seems to be the most promising, and would allow for unbiased and accurate determination of TBA, whilst producing analytical data that could be easily incorporated in electronic batch reports. However, at present time, this equipment is not commercially available, and the cost is unknown. 

Iodoplatinate is a well-known staining solution for visualizing tertiary amines, and is used as the standard Pharm Eur. method for determining the Kryptofix ^®^ 2.2.2 (Kryptofix) content in [^18^F]FDG [1,7]. In the literature, there are several descriptions of iodoplatinate being used to visualize quaternary ammonium compounds [8,9]. However, one of the problems with iodoplatinate-stained TLC plates is that they yield a false-negative result if the formulation to be tested contains an antioxidant stabilizer, such as sodium ascorbate, which is frequently used in ^18^F-radiopharmaceutical formulations [10]. As none of the methods described in the literature yield satisfactory results for determining the presence of TBA in all common ^18^F-radiopharmaceutical formulations, we decided to explore whether TBA content could be determined by the same general methodology as described in Pharm Eur. for Kryptofix, and to mend the antioxidant problem, thereby producing a standard methodology that would work for every ^18^F-radiopharmaceutical. We herein report a successful analysis of limit level TBA in ^18^F-radiopharmaceuticals, using easily available iodoplatinate-stained TLC plates, commonly in use in most PET-tracer quality control labs. The test is also found to work for antioxidant containing ^18^F-radiopharmaceuticals. 

## 2. Results

**Figure 1:**
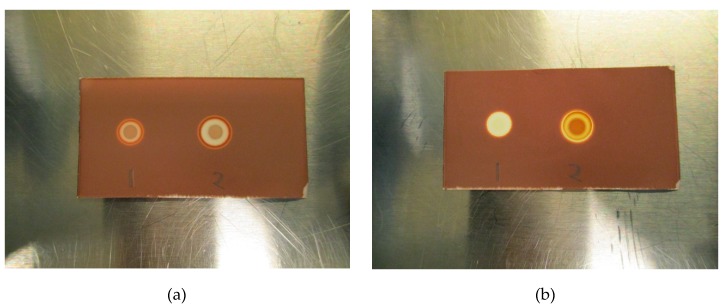
**(a**) Initial test without antioxidant: (1) Tetrabutylammonium (TBA) standard (0.26 mg/mL), (2) TBA standard (0.26 mg/mL) + water. (**b**) Initial test with antioxidant: (1) TBA standard (0.26 mg/mL), (2) TBA standard (0.26 mg/mL) + H_2_O_2_.

**Figure 2:**
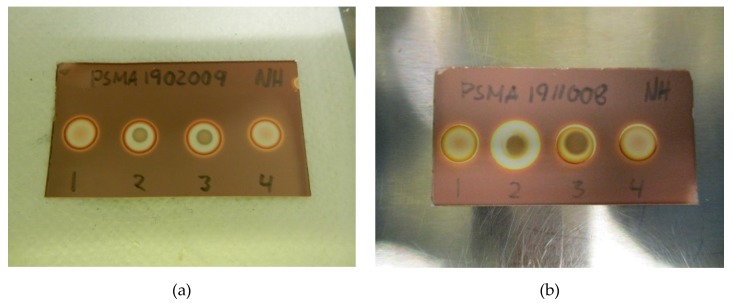
(**a**) Specificity without antioxidant: (1) [^18^F]PSMA-1007 + water, (2) TBA standard (0.26 mg/mL) + [^18^F]PSMA-1007, (3) TBA standard (0.26 mg/mL) + water, (4) matrix + water. (**b**) Specificity with antioxidant: (1) [^18^F]PSMA-1007 + H_2_O_2_, (2) TBA standard (0.26 mg/mL) + [^18^F]PSMA-1007 + H_2_O_2_, (3) TBA standard (0.26 mg/mL) + H_2_O_2_, (4) matrix + H_2_O_2_.

Initial testing showed that a 2.5 µL application of TBA (0.26 mg/mL) produces a brown spot in the middle of a white circle in contact with iodoplatinate-stained TLC plates (Figure 1a, (1)). The addition of 2.5 µL of water on top of the same TBA spot made the spot appear brighter, thereby increasing the detection limit (Figure 1a, (2)). When an antioxidant was present in the ^18^F-radiopharmaceutical formulation, a bright white spot would occur on the iodoplatinate plate, without any coloration due to TBA (0.26 mg/mL), despite TBA being present (Figure 1b, (1)). By adding 2.5 µL of hydrogen peroxide (H_2_O_2_) on top of the false-negative TBA spot around 30 s after the initial application of the TBA spot, the brown spot from TBA complexing with iodoplatinate was revealed, with an orange and white-colored circle around it as a result of the oxidation (Figure 1b, (2)). Consequently, it was found that the time it took for the brown coloration in the middle of the spot to appear depended both on the amount of antioxidant present in the formulation and the concentration of H_2_O_2_ used. For the maximum amount of sodium ascorbate (20 mg/mL) in [^18^F]PSMA-1007 that was tested, it was found that a 1% (w/w) solution of H_2_O_2_ was sufficient to produce a distinct, brown and concentric spot in about one minute (Figure 2b). Some differences in the brown color of the spot depending on the matrix with antioxidant added were observed. TBA spots in the [^18^F]FDOPA matrix were a lighter shade of brown than spots from the [^18^F]PSMA matrix, this however, did not affect the visibility. To be certain that these effects are true and reliable it was decided to validate the method according to ICH Q2(R1) [11]. For a limit test, the specificity and limit of detection (LoD) must be proven. To determine that the method was specific, the following criteria needed to be fulfilled:

- Spots from the matrix and the ^18^F-radiopharmaceutical sample are identical.
- Spots from TBA can be clearly distinguished from spots from the matrix and the ^18^F-radiopharmaceutical sample.
- Spots from the matrix spiked with TBA and spots from the ^18^F-radiopharmaceutical sample spiked with TBA are identical.
- Spots from TBA in the allowable pH range for the ^18^F-radiopharmaceutical in question must be identical.

**Figure 3:**
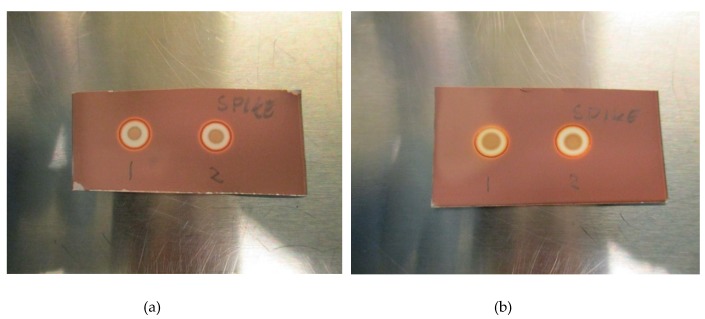
(**a**): Spike without antioxidant: (1) Matrix spiked with TBA, (2) [^18^F]PSMA-1007 spiked with TBA. (**b**): Spike with antioxidant: (1) Matrix spiked with TBA, (2) [^18^F]PSMA-1007 spiked with TBA.

The LoD was determined by diluting the TBA solution until a concentration where the spots could no longer be identified as a distinct brown coloration. All validation experiments were run three times (n = 3). The experiments with hot ^18^F-radiopharmaceuticals were run immediately after release of the quality control sample following production. The TBA dilution and pH experiments were prepared as “cold” solutions, i.e., without hot [^18^F]PSMA, [^18^F]FLT or [^18^F]FDOPA. This was done to protect the operators from unnecessary radiation, according to the ALARA (as low as reasonably achievable) principle. Due to low concentrations of actual [^18^F]fluorine compounds in the final solution, it is highly unlikely that these compounds will interfere with the spot test, and as a result, the main potential influencing factors are determined to be matrix excipients like buffers and stabilizers. In order to prove this to be true, hot [^18^F]PSMA was spiked with TBA and a spot from the hot sample was compared visually with a spot from a cold [^18^F]PSMA matrix (Figure 3a,b). 

## 3. Discussion

The purpose of the study was twofold. It had to show that the iodoplatinate plates were suitable for the analysis of TBA and to find a solution for the problem with ascorbate/antioxidant containing matrixes. The false-negative effect that the ascorbate induces is thought to be due to ascorbate reducing the platinum (IV) species to a platinum (II) species. By the addition of H_2_O_2_, the platinum (II) species is oxidized back to a platinum (IV) species, which can then form a colored complex with TBA. Findings to support this notion were observed when an ascorbate solution (5 mg/mL) was added dropwise to the standard iodoplatinate solution, until a color change from deep purple to golden yellow occurred. The consequent dropwise addition of a H_2_O_2_ solution (1% w/w) resulted in a rapid color change back to the deep purple color of the original iodoplatinate solution. 

Figure 2a,b show a typical spot test for TBA, both with and without the ascorbate-containing matrix. The spot test is constructed in an identical manner to the spot test of Kryptofix in [^18^F]FDG, i.e., spot (1) is the radiotracer sample, spot (2) is the sample + reference standard, to show that there is no interference with the two, spot (3) is the reference standard in a concentration representing the acceptance limit. Spot (4) is water or matrix, representing a blank test. From the test it is evident that spot (2) and (3) containing a reference standard are clearly and distinctly different from spot (1) and (4), unequivocally showing the efficiency of the test. Figure 3a, b show that the spiked sample and spiked matrix yield identical spots, and therefore that [^18^F]PSMA-1007 has no interference on the test.

**Figure 4:**
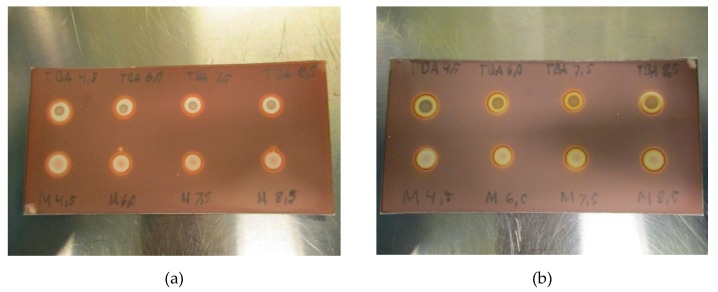
(**a**): TBA and matrix spots pH-range 4.5–8–5 for [^18^F]PSMA-1007 without antioxidant and (**b**): TBA and matrix spots pH-range 4.5–8–5 for [^18^F]PSMA-1007 with antioxidant.

Due to the nature of the spot test and the dependence of TBA creating a colored complex with platinum, pH might be of relevance. To examine the effect of pH on the test, the matrices with pH varying from 4.5 to 8.5 were prepared and analyzed. As depicted in Figure 4a,b, no effect was found from changes in pH, neither with nor without ascorbate in the matrix, thus, TBA spots are unaffected by pH in the allowable range.

**Figure 5:**
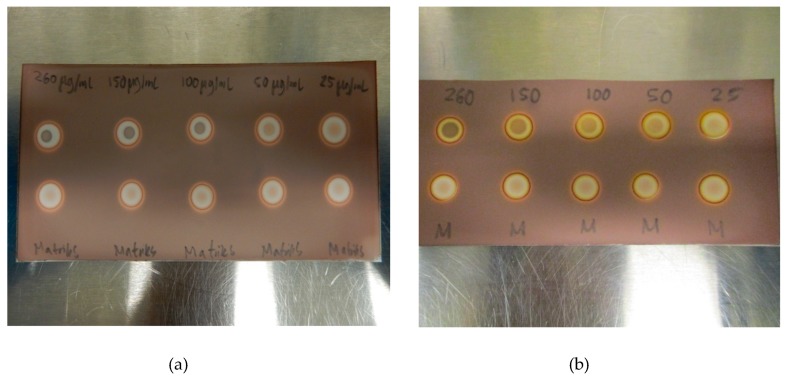
(**a**) LoD determination for TBA in the [^18^F]PSMA-1007 matrix without antioxidant and. (**b**) LoD determination for TBA in the [^18^F]PSMA-1007 matrix with antioxidant.

In order to examine the range of the spot test, a linearity test was constructed. Five different concentrations of TBA in the matrix, ranging from 25 µg/mL to 260 µg/mL, were prepared, both with and without ascorbate. As expected, the difference between the TBA-containing matrix and the blank matrix became smaller with decreasing concentrations of TBA, and at 25 µg/mL, the trained eye might see the difference, but it is no longer striking. Three different QC operators, not familiar with the test, agreed that 50 µg/mL of TBA was visually distinct, thus, LoD was found to be around 50 µg/mL for all the tested matrix solutions (Figure 5a,b). There was a clear and distinct difference in the intensity of the spots depending on the concentration of TBA. No false-positive or false-negative results were observed during validation or later during routine usage of the method. 

**Figure 6:**
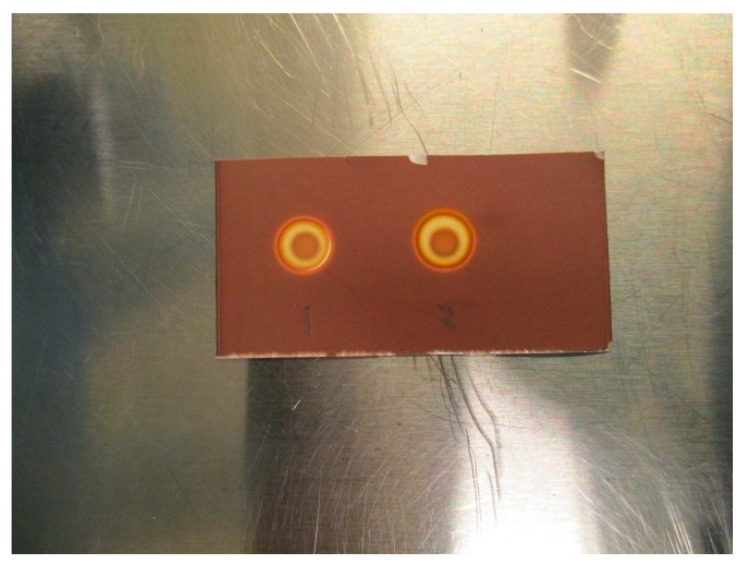
(1) TBA standard (0.26 mg/mL) + H_2_O_2_ (1% w/w), (2) TBA standard (0.26 mg/mL) + H_2_O_2_ (3% w/w).

The concentration of antioxidant present in the matrix was found to only have one significant effect on the spot test, and this effect was that higher concentrations increased the time it took for the brown coloration to appear after the addition of H_2_O_2_ (1% w/w). As an example, when the concentration was 5 mg/mL, it took about 30 s for coloration, while at a concentration of 20 mg/mL, it took a little over a minute. Higher concentrations of H_2_O_2_ sped up the coloration process without affecting the test in any other way (Figure 6). Therefore, it is possible to increase H_2_O_2_ concentration if necessary.

**Figure 7:**
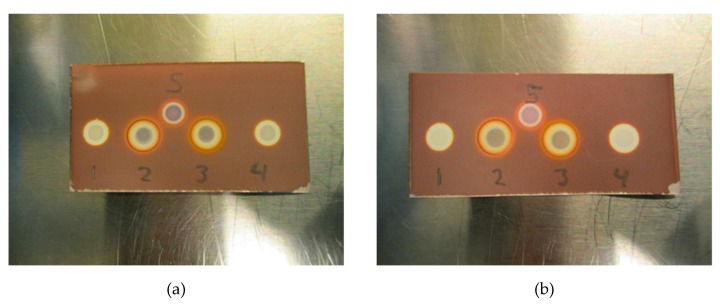
(**a**) Test with Kryptofix. (1) Kryptofix (0.22 mg/mL in 20 mg/mL ascorbate matrix), (2) Kryptofix (0.22 mg/mL in 20 mg/mL ascorbate matrix) + H_2_O_2_, (3) Kryptofix (0.22 mg/mL in 5 mg/mL ascorbate matrix) + H_2_O_2_, (4) Kryptofix (0.22 mg/mL in 5 mg/mL ascorbate matrix), (5) Kryptofix (0.22 mg/mL in matrix without ascorbate). (**b**) Test with Kryptofix at lower concentration. (1) Kryptofix (0.11 mg/mL in 20 mg/mL ascorbate matrix), (2) Kryptofix (0.11 mg/mL in 20 mg/mL ascorbate matrix) + H_2_O_2_, (3) Kryptofix (0.11 mg/mL in 5 mg/mL ascorbate matrix) + H_2_O_2_, (4) Kryptofix (0.11 mg/mL in 5 mg/mL ascorbate matrix), (5) Kryptofix (0.11 mg/mL in matrix without ascorbate).

Initial testing (Figure 7a,b) with Kryptofix (0.22 or 0.11 mg/mL) in a PBS matrix with either 20 mg/mL ascorbate or 5 mg/mL ascorbate indicates that the test also works for determining if Kryptofix is present in an antioxidant containing a ^18^F-radiopharmaceutical formulation. It is evident from Figure 7a,b that ascorbate also affects the Kryptofix spot test, however, the addition of H_2_O_2_ (1% w/w) in a similar manner as for the TBA spot test, remedies the issue. 

A simple test for determining if TBA is present in ^18^F-radiopharmaceuticals has been developed and validated. The test is fast (less than five minutes), consistent and robust. Matrix effects were only observed when the final formulation contained an antioxidant, and a solution to reverse the matrix effect was developed. The test is similar to the spot test for Kryptofix described in Pharm Eur. and it is therefore easy to implement in laboratories already running that test for routine [^18^F]FDG productions. The test has successfully been implemented in our quality control facility. 

## 4. Materials and Methods 

### 4.1. General

Chemicals of the highest available grade were purchased from Sigma-Aldrich Norway AS (Oslo, Norway) or VWR International AS (Oslo, Norway). 

Specified chemicals with article number and manufacturer in brackets:

Tetrabutylammonium hydroxide 30-hydrate (≥ 99.0%) (Sigma-Aldrich, 86859)

Hexachloroplatinic (IV) acid hexahydrate (~40% Pt) (Merck, 8073400005)

Potassium iodide (≥ 99.5%) (Sigma-Aldrich, 30315)

Silica gel 60 F_254_ TLC plates 50 × 100mm (Merck, 116834)

### 4.2. Production of [^18^F]Fluoride

No-carrier-added [^18^F]fluoride was produced on a PETtrace cyclotron 840 (GE Healthcare, Uppsala, Sweden) via ^18^O(p,n)^18^F nuclear reaction. 2.8 mL enriched [^18^O]water (>97%, Rotem, Be’er Sheva, Israel) was irradiated with 16.5 MeV protons in a niobium target body with Havar ^®^ foils. 

### 4.3. ^18^F-Radiopharmaceuticals

[^18^F]FLT was produced on a FASTlab 2 synthesizer (GE Healthcare, Oslo, Norway) with cassettes and reagent kits from GE Healthcare (Oslo, Norway), yielding 22 mL of product in water for injection and ethanol (~10%) with an average pH of 5.5–6.0. 0.8 mL of 0.14M tetrabutylammonium hydrogen carbonate was used as a source for TBA in the synthesis. The average activity of the product was 600 MBq/mL.

[^18^F]FDOPA was produced on a FASTlab 2 synthesizer (GE Healthcare, Oslo, Norway) with cassettes and reagent kits from ABX (Radeberg, Germany), through nucleophilic pathway, yielding 28 mL of product in a phosphate buffer containing ascorbic acid, ethylenediaminetetraacetic acid disodium and ethanol (specification limit 10%, average content ~ 3%), with an average pH of 5.0–5.5. In this study, 0.75 mL of 0.075M tetrabutylammonium hydrogen carbonate was used as a source for TBA in the synthesis. The average activity of the product was 200 MBq/mL. 

[^18^F]PSMA-1007 was produced on a Mosaic-RS synthesizer (Neptis, Philippeville, Belgium) with cassettes and reagent kits from ABX (Radeberg, Germany), yielding 20 mL of product in phosphate-buffered saline (PBS) containing ethanol (~10%), with an average pH of 7.4. For testing antioxidant effects, 400 mg sodium ascorbate was added to the final product elution vial to yield a concentration of 20 mg/mL sodium ascorbate in the final product for select productions of [^18^F]PSMA-1007. Then, 1.05 mL of 0.075M tetrabutylammonium hydrogen carbonate was used as a source for TBA in the synthesis. The average activity of the product was 1400 MBq/mL. 

### 4.4. Preparation of Standard Solutions 

TBA in [^18^F]PSMA-1007 matrix without antioxidant stabilizer: TBA stock solution (10 mg/mL) was prepared by dissolving 330 mg tetrabutylammonium hydroxide 30-hydrate in 10 mL matrix solution (PBS (pH 7.4) and ethanol (10% V/V)). Standard solutions of 0.5, 0.26, 0.150, 0.100, 0.05 and 0.025 mg/mL TBA were prepared by diluting 500, 260, 150, 100, 50 and 25 µL of stock solution to 10 mL with matrix solution. [^18^F]PSMA-1007 spike solution was prepared by adding 26 µL TBA stock solution (10 mg/mL) to 974 µL [^18^F]PSMA-1007 solution, resulting in a concentration of 0.26 mg/mL TBA. The solutions for testing pH dependency were prepared by adjusting the pH of the matrix by adding 1M NaOH or 1M HCl to yield matrix solutions with pH 4.5, 6.0, 7.5 and 8.5. 

TBA in [^18^F]PSMA-1007 matrix with antioxidant stabilizer: TBA stock solution (10 mg/mL) was prepared by dissolving 330 mg tetrabutylammonium hydroxide 30-hydrate in 10 mL matrix solution (PBS (pH 7.4), ethanol (10% (V/V) and sodium ascorbate (20 mg/mL))). Standard solutions of 0.5, 0.26, 0.150, 0.100, 0.05 and 0.025 mg/mL TBA were prepared by diluting 500, 260, 150, 100, 50 and 25 µL of stock solution to 10 mL with matrix solution. 

TBA in [^18^F]FLT matrix: [^18^F]FLT formulation does not contain any other excipients than ethanol diluted in water for injection. Therefore, it was unnecessary to test this solution for matrix effects. The method was validated by spiking cold [^18^F]FLT batches with TBA and verifying the method with warm [^18^F]FLT batches. Then, [^18^F]FLT spike solution was prepared by adding 26 µL TBA stock solution (10 mg/mL) to 974 µL [^18^F]FLT solution, resulting in a concentration of 0.26 mg/mL TBA. 

TBA in [^18^F]FDOPA matrix: TBA stock solution (10 mg/mL) was prepared by dissolving 330 mg tetrabutylammonium hydroxide 30-hydrate in matrix solution (Phosphate buffer (pH 6.1), ascorbic acid (1 mg/mL), ethylenediaminetetraacetic acid disodium salt dihydrate (1.5 mg/mL) and ethanol (10% V/V)). Standard solutions of 0.26, 0.150, 0.100, 0.05 and 0.025 mg/mL TBA were prepared by diluting 260, 150, 100, 50 and 25 µL of stock solution to 10 mL with matrix solution. [^18^F]FDOPA spike solution was prepared by adding 26 µL TBA stock solution (10 mg/mL) to 974 µL [^18^F]FDOPA solution, resulting in a concentration of 0.26 mg/mL TBA. The solutions for testing pH dependency were prepared by adjusting the pH of the matrix by adding 1M NaOH or 1M HCl to yield matrix solutions with pH 4.0, 4.5, 5.0 and 5.5. 

H_2_O_2_ solution: (1% w/w) was prepared by weighing in 3.33 g H_2_O_2_ (3% w/w) and diluting to 10 g with deionized water. 

Preparation of the iodoplatinate indicator strips: Iodoplatinate reagent was prepared by mixing 2.5 mL hexachloroplatinic (IV) acid hexahydrate in water (5% w/v) with 22.5 mL potassium iodide in water (10% w/v) and diluting with an additional 50 mL of water. The spot test plates were prepared by immersing TLC plates (50 × 100 mm) in the iodoplatinate reagent solution for 10 s, applying a gentle stream of air to dry off excess reagent and leaving to dry overnight in a vented container at room temperature, protected from light. For routine quality control spot test use, the plates are cut into four identical sized pieces (50 × 25 mm). 

### 4.5. Test Protocol for Routine Quality Control

#### 4.5.1. TBA without Antioxidant Stabilizer

An iodoplatinate-stained TLC plate is spotted with 2.5 µL [^18^F]PSMA-1007 or [^18^F]FLT (1), and 0.26 mg/mL TBA twice ((2) and (3)) and also on the matrix (4) to give four spots (Figure 2a). After 1 min of air drying, 2.5 µL of water was applied on top of previous spots (1, 3 and 4) while 2.5 µL [^18^F]PSMA-1007 or [^18^F]FLT was applied on top of the second TBA spot (2). After 1 min of air drying the plate was visually analyzed for TBA content.

#### 4.5.2. Acceptance Criteria:

System suitability

- Spot (2) and (3) are similar in that they consist of a brown circle in the center with a white circle around. The intensity of the brown circle is proportional to the concentration of the impurity (TBA).
- Spot (4) consists of a pink area in the middle of a white circle.
- Spot (4) is clearly different from spot (2) and (3).

Limit

The central part of spot (1) is no more intense than the central part of spot (2) and (3).

#### 4.5.3. TBA with Antioxidant Stabilizer

An iodoplatinate-stained TLC plate is spotted with 2.5 µL [^18^F]PSMA-1007 or [^18^F]FDOPA(1), 0.26 mg/mL TBA two times (2) and (3) and matrix (4) to give four spots (Figure 2b). After 1 min of air drying 2.5 µL of H_2_O_2_ solution was applied on top of previous spots (1, 3 and 4) while 2.5 µL [^18^F]PSMA-1007 or [^18^F]FDOPA was applied on top of the second TBA spot (2), followed by 2.5 µL H_2_O_2_. After 1 min of air-drying, the plate was visually analyzed for TBA content.

Acceptance criteria:

System suitability

- Spots (2) and (3) are similar in that they consist of a brown circle in the center with an orange and white circle around it. The intensity of the brown circle is proportional to the concentration of the impurity (TBA).
- Spot (4) consists of a pink area in the middle of an orange and white circle.
- Spot (4) is clearly different from spot (2) and (3).

*Limit*

The central part of spot (1) is no more intense than the central parts of spots (2) and (3).
